# Estimate of incidence and cost of recreational waterborne illness on United States surface waters

**DOI:** 10.1186/s12940-017-0347-9

**Published:** 2018-01-09

**Authors:** Stephanie DeFlorio-Barker, Coady Wing, Rachael M. Jones, Samuel Dorevitch

**Affiliations:** 1 Division of Environmental and Occupational Health Sciences, School of Public Health, University of Illinois, 2121 W. Taylor, Chicago, Illinois USA; 2 School of Public Health and Environmental Affairs, Indiana University, Bloomington, Indiana USA; 3 Institute for Environmental Science and Policy, University of Illinois, Chicago, Illinois USA

**Keywords:** Water recreation, Economic burden, National estimate

## Abstract

**Background:**

Activities such as swimming, paddling, motor-boating, and fishing are relatively common on US surface waters. Water recreators have a higher rate of acute gastrointestinal illness, along with other illnesses including respiratory, ear, eye, and skin symptoms, compared to non-water recreators. The quantity and costs of such illnesses are unknown on a national scale.

**Methods:**

Recreational waterborne illness incidence and severity were estimated using data from prospective cohort studies of water recreation, reports of recreational waterborne disease outbreaks, and national water recreation statistics. Costs associated with medication use, healthcare provider visits, emergency department (ED) visits, hospitalizations, lost productivity, long-term sequelae, and mortality were aggregated.

**Results:**

An estimated 4 billion surface water recreation events occur annually, resulting in an estimated 90 million illnesses nationwide and costs of $2.2- $3.7 billion annually (central 90% of values). Illnesses of moderate severity (visit to a health care provider or ED) were responsible for over 65% of the economic burden (central 90% of values: $1.4- $2.4 billion); severe illnesses (result in hospitalization or death) were responsible for approximately 8% of the total economic burden (central 90% of values: $108- $614 million).

**Conclusion:**

Recreational waterborne illnesses are associated with a substantial economic burden. These findings may be useful in cost-benefit analysis for water quality improvement and other risk reduction initiatives.

**Electronic supplementary material:**

The online version of this article (doi: 10.1186/s12940-017-0347-9) contains supplementary material, which is available to authorized users.

## Background

Surface water recreation in lakes, ponds, rivers, and oceans is popular in the US. According to the National Survey on Recreation and Environment (NSRE), over 61% (142 million people) of the US population over 16 years of age used waterbodies to participate in a range of non-motorized water sports like swimming, kayaking, and canoeing [1].

The enjoyment people get from surface water recreation is tempered by the risk of illness from exposure to polluted water. Water recreators have a higher incidence of acute gastrointestinal illness (AGI) and non-enteric illnesses, including respiratory, ear, eye, and skin symptoms than non-water recreators [2–6]. Sporadic illnesses from water recreation often are mild and identified through epidemiologic studies [3, 7]. In contrast, outbreaks among surface water recreators are documented in the Waterborne Disease and Outbreak Surveillance System (WBDOSS) annually. From 2000 to 2010 WBDOSS recorded 102 outbreaks associated with untreated recreational water (excluding swimming pools and water parks), and more than 1559 cases of illness. WBDOSS is a passive outbreak surveillance system. Members of the public, clinicians, or clinical microbiology personnel are to notify health department personnel about suspected outbreaks. Health department personnel investigate to verify whether an outbreak has occurred and whether it is linked to water recreation. Recognized outbreaks constitute an unknown fraction of all waterborne disease outbreaks that occur [8].

To date, the literature does not describe the number of illnesses attributable to surface water recreation in the US or the costs of such illnesses per year. Without these estimates, costs of improved public health protections, beach monitoring and notification programs, or beach remediation efforts cannot be put into context. Assessments of economic burden from illnesses due to water recreation have been published in recent years though they are limited either in terms of data availability, a focus on a specific location [9], or focus on costs associated with hospitalization of illnesses thought to be transmitted by water, but not specifically through recreational water exposure [10]. We have recently described the severity [11] and costs [12] associated with sporadic cases of AGI in two large cohort studies of water recreation. Costs of AGI attributable to water recreation per 1000 recreators, ranged between $338 and $1681 for incidental-contact recreation (paddling, motor-boating, and fishing) and between $425 and $2743 for swimming/wading per 1000 recreators. Our primary objective in the present study was to construct an estimate of the national economic burden of surface water recreation in the US for sporadic and outbreak-associated illnesses.

## Methods

### Overview

To estimate the annual number of cases of illness (AGI, respiratory, ear, eye, and skin) attributable to surface water recreation, we first estimated the number of people who engage in surface water recreation annually. We combined estimates of the size of the water recreator population with estimates of rates of the occurrence of illness and the fraction of illnesses attributable to water recreation from cohort studies of swimming and incidental-contact recreation to form estimates of the number of sporadic illness that occur annually due to water recreation. Two cohort studies used to estimate sporadic cases of recreational waterborne illness in the US included the National Environmental and Epidemiological Assessment of Recreational Water (NEEAR, which addressed swimming) and the Chicago Health, Environmental Exposure, and Recreation Study (CHEERS, which addressed incidental-contact recreation, such as paddling, motor-boating, and fishing). NEEAR evaluated health outcomes among approximately 26,000 beachgoers at freshwater and marine water beaches in six states [4–6] between 2003 and 2009. CHEERS evaluated similar health outcomes of incidental-contact water recreation, enrolling over 11,000 participants at 39 locations in the Chicago, Illinois area between 2007 and 2009 [3]. These studies evaluated the health impacts of water recreation at locations impacted by human fecal pollution, among a large number of water recreators.

We defined illnesses in the analysis as being mild, moderate, or severe. Mild illnesses were defined as not having contact with a health care provider (HCP), while moderate illnesses were defined has having contact with a HCP, whether in an outpatient facility, or an emergency department (ED). Rates of mild and moderate illness were estimated using the two cohort studies. Severe illnesses were defined as illnesses resulting in hospitalization or death, and were estimated using WBDOSS outbreak surveillance data. Assumptions of FoodNet surveillance system researchers were used to account for underdiagnosis and underreporting of cases of severe illness in the outbreak surveillance system [13]. Costs for all levels of severity were estimated based on the use of over-the-counter (OTC) medication, prescription medication, and time missed from work. Moderate illnesses also included costs associated with relevant HCP or ED visits. Severe illnesses, also included costs of hospitalization (rates were estimated based on both WBDOSS outbreak data and published studies (Additional file 1: Table S1), death (using value of a statistical life (VSL) [14], as well as costs associated with other health consequences which developed due to the primary illness (sequelae). By multiplying the estimated number of cases of illness attributable to water recreation (recreation events × attributable risk) by the distribution of costs we arrived at a range of cost estimates for each illness type (mild, moderate, or severe). Monte Carlo simulation calculated 100,000 realized values of the potential cost of surface water recreation, allowing us to estimate the mean and central 90% of values (C90) (5th and 95th percentile of the distribution) of costs for mild and moderate gastrointestinal, respiratory, eye, ear, skin conditions, as well as pathogen-specific severe illnesses attributable to water recreation.

### Data sources: Number of water recreation events

The number of swimmers/waders, paddlers, motor-boaters, and anglers per year in the US were estimated using the proportion of the non-institutionalized US population over 16 years of age who engage in water recreation each year [1]. These proportions, combined with the total US non-institutionalized population in 2007, estimated by the US Census [15], yield the total number of water recreators in the US over 16 years. Estimates of the total number of water recreators 16 years and under were estimated based on the proportion of persons 16 and under, relative to the number of those over age 16 by activity in two studies of water recreation [3, 4, 6]. The estimated number of recreation events was calculated using national estimates of days of water recreation per water recreator by recreational activity [1].

### Mild and moderate illness occurrence and severity

NEEAR and CHEERS data demonstrate that among participants who developed AGI, over 50% took OTC medications, 40% stayed home from work, school, or missed daily activities, almost 20% were in contact with a HCP, 10% took prescription medications, and 1% went to an ED [11]. Costs of AGI varied among water recreators, depending on whether illness resulted in lost activities (such as work) and the extent to which individuals with AGI utilized the healthcare system [12]. In addition to AGI, we also evaluated the occurrence and costs of ear, eye, skin, and respiratory symptoms, by calculating the attributable risks based on the two previous epidemiology studies (Additional file 1: Table S2). Severity and costs of these non-AGI illnesses were evaluated similarly (See Additional file 1) as was done previously [12].

### Severe illness occurrence and severity

To estimate the number of hospitalizations and deaths due to each pathogen, we applied pathogen-specific hospitalization and death rates among laboratory-confirmed illnesses (see Additional file 1: Table S1). Recognized outbreaks represent an unknown fraction of all outbreaks, and underreporting and underdiagnosis are threats to the accuracy of waterborne disease outbreak surveillance data. To mitigate underreporting bias, we used estimates of underreporting rates from validation studies to construct adjusted illness counts, which were then used to estimate the number of hospitalizations and deaths. In a study of foodborne outbreaks of gastrointestinal infections, Scallan et al. [13] found that for every case of illness identified during an outbreak, approximately 25.5 other cases were estimated to occur without being captured by the outbreak surveillance system. We used this multiplier of 25.5 to correct for the underreporting and underdiagnoses of pathogen-specific recreational waterborne illnesses under the assumption that generally the same types and severity of illnesses would be underreported and underdiagnosed at similar rates whether the source of the illness was waterborne or foodborne. Cases of vibriosis linked to surface water recreation identified in WBDOSS include outbreaks as well as single cases that occurred outside recognized outbreaks [16]. As a result, numbers of vibriosis cases are thought to be better estimated; and only an underreporting multiplier of 1.1 developed for foodborne gastrointestinal illness [13] was applied. Once the number of outbreak-related cases were estimated, we then approximated the proportion of these cases which would be hospitalized or would result in death, since our severe illness category only included hospitalizations and deaths due to recreational waterborne illness (Additional file 1: Table S5 and S10). No multipliers were used for cases of primary amoebic meningoencephalitis due to *Naegleria fowleri*, since the illness is nearly 100% fatal and is unlikely to go undiagnosed and unrecognized [17]. Hospitalization data and death data (distinct from counts of the number of cases in communities) can also underestimate the number of cases caused by specific pathogens. Therefore, to account for underdiagnosis and/or underreporting, namely, we doubled the number of hospitalizations and deaths [13, 18] realizing this multiplier is subject to uncertainty.

### Economic burden estimation

Health-related costs among the projected number of ill water recreators were summarized for mild (no contact with HCP), moderate (contact with HCP), and severe (hospitalization or deaths identified from WBDOSS) waterborne illnesses using methods common in studies estimating total economic burden of ill health [19, 20]. Total costs associated with each outcome (*i* = [mild, moderate, severe]) were estimated according to Eq. 1, using the total cost of OTC medications (*OTC*_*i*_), prescription medications (*Rx*_*i*_), HCP visits (*HCP*_*i*_), ED visits (*ED*_*i*_), hospitalizations (*Hospital*_*i*_), sequelae (*Sequelae*_*i*_), indirect costs resulting from missing time from work *(Productivity*_*i*_), as well as costs associated with mortality, using the Value of a Statistical Life (*VSL*_*i*_) [14] due to illness. All costs were adjusted for inflation to 2007 dollars (the year when both NEEAR and CHEERS overlapped), using relevant consumer price index for medical care commodities (medications) and medical care services (medical and hospital services) [21]. Cost assumptions and data sources are described in the (Additional file 1: Table S1). Costs for ED visits or hospitalizations were estimated from the Nationwide Emergency Department Sample (NEDS) and Nationwide Inpatient Sample (NIS) respectively, aggregated by the Agency for Healthcare Research and Quality (AHRQ), by ICD-9-CM (Additional file 1: Table S3). Total excess costs per case due to the development of with sequelae were estimated for the following specific illnesses: Guillain-Barré syndrome (*Campylobacter*) [19, 22], hemolytic-uremic syndrome with or without end stage renal disease (*E. coli 0157H7*), [19, 20, 23], and reactive arthritis (*Campylobacter*, *Shigella*) [19, 24, 25].

1 $$ {Cost}_i={OTC}_I+{Rx}_i+{HCP}_i+{ED}_i+{Hospital}_i+{Sequelae}_i+{Productivity}_i+{VSL}_i $$

Rather than using point estimates to develop an estimate of the total economic burden, we used a stochastic approach using a Monte Carlo simulation. This simulation is an effort to account for inherent randomness in the many estimates involved in the analysis. A single run of the simulation works by randomly drawing a value from each of the distributions (Additional file 1: Tables S4-S8) from means and other moments reported in the literature or estimated from the cohort studies. With the selected parameters, numbers of ill recreators and costs are calculated and then stored. This process was repeated 100,000 times to yield an estimated mean costs and the 5th and 95th percentile of the distribution, or the central 90% of values (C90) around the mean. The simulation was conducted using Crystal Ball version 11.1.

## Results

Approximately 4.04 billion surface water recreation events were estimated to occur annually in the US. People who engage in many of these activities tend to do so multiple times annually [1]. The largest number of person-days of water recreation are accounted for by swimming (1.9 billion, 47.7%), followed by fishing (1.1 billion, 27.3%) (Table 1).

**Table 1:** Estimated percent of the population that engages in each activity, and the number of water recreators and person-days of water activity

Activity	% of population 16 and over^a^	Millions of Recreators 16 and over^b^ (C90)	% of population under 16^c^	Millions of recreators under 16^d^ (C90)	Mean days per recreator (C90)^a^	Millions of person days (C90)
Swimming	41.5 (40.9–42.0)	96. 9 (96.1–97.7)	88.8 (88.0–89.5)	56.3 (55.8–56.7)	12.6 (12.3–12.9)	1928.9 (1882.2–1976.9)
Kayaking	6.0 (5.8–6.3)	14.1 (13.7–14.4)	1.7 (1.6–1.7)	1.1 (1.0–1.1)	5.6 (5.3–6.0)	84.4 (78.5–91.2)
Rowing	4.0 (3.8–4.2)	9.3 (9.4–9.3)	1.3 (1.2–1.3)	0.8 (0.8–0.8)	5.5 (5.0–6.0)	55.8 (54.1–57.6)
Canoeing	9.7 (9.4–10.0)	22.7 (22.2–23.1)	4.9 (4.8–5.0)	3.1 (3.1–3.2)	4.7 (4.5–4.9)	121.1 (115.6–126.8)
Motor boating	23.4 (22.9–23.9)	54.7 (53.9–55.4)	12.7 (12.5–12.8)	8.0 (7.9–8.1)	11.9 (11.2–12.3)	745.8 (719.0–772.7)
Fishing ^e^	23.7 (23.2–23.9)	55.3 (54.6–56.1)	32.3 (31.7–32.6)	20.4 (20.1–20.6)	14.6 (14.1–15.2)	1105.1 (1057.9–1152.3)
Total						4.041

^a^Cordell 2012 [1]

^b^ Estimated based of US noninstitutionalized population 16 and over (233.5 million) in 2007 [15]

^c^ Estimated number of recreators under 16/Estimated US population under 16 (63.3 million in 2007) [15]

^d^ Estimated based on the proportion of children under 16 in NEEAR and CHEERS and the number of adult recreators in the US population

^e^ Assuming warm water fishing

Epidemiologic data indicates risk for developing AGI symptoms attributable to swimming/wading of approximately 15 cases per 1000 recreators (Table 2). The risk of AGI illness attributable to fishing is approximately 15 per 1000, while the risk attributable to other incidental-contact water recreation activities is 6 per 1000. The attributable risks of respiratory, eye, ear, and skin symptoms varied by type of water recreation. Given the number of water recreation events (Table 1), and the activity-specific attributable risks (Table 2), we estimate that of the 50 million sporadic cases (outside of recognized outbreaks) of AGI illness attributable to water recreation, 29 million (57%) are attributable to swimming and 16.5 million (32%) are attributable to fishing (Table 3). Approximately 10 million cases each of respiratory, eye, ear, and skin illness attributable to water recreation occur annually. These cases are almost entirely in the context of swimming, with the exception of eye symptoms, which primarily occur in the context of motor-boating. The vast majority of sporadic illnesses are mild in the sense that they do not result in contact with a HCP. Respiratory, eye, and ear symptoms were more likely to result in moderate illness involving HCP contact (Table 3). According the epidemiologic data, adults (aged 20–54) tend to experience the majority of mild illnesses, while children (0–10) experience the majority of moderate illnesses (Additional file 1: Table S9). It is less clear how age effects those with severe illness who are hospitalized or who die as this information generally not publicly available. Based on outbreak data, we estimate that between 333 and 1696 hospitalizations and 16–67 deaths occur annually due to recreational waterborne illness (Additional file 1: Table S10).

**Table 2:** Attributable risks (95% CI) for the development of symptoms among water recreators, by activity

	Incidental-Contact: Fishing^a^			Other Incidental-Contact^b^			Swimming/Wading^c^		
	Probability of illness if exposed water/fish/bait (95% CI)	Probability of illness if unexposed (95% CI)	Attributable Risk (95% CI)	Probability of illness if exposed to water (95% CI)	Probability of illness if unexposed (95% CI)	Attributable Risk (95% CI)	Probability of illness if exposed to water (95% CI)	Probability of illness if unexposed (95% CI)	Attributable Risk (95% CI)
AGI	0.053 (0.039, 0.065)	0.038 (0.031, 0.046)	0.015 (−0.003, 0.030)	0.040 (0.036, 0.046)	0.034 (0.028, 0.040)	0.006 (−0.002, 0.014)	0.040 (0.036, 0.043)	0.025 (0.022, 0.029)	0.015 (0.010, 0.020)
Respiratory	--^d^	--^d^	–	0.182 (0.171, 0.192)	0.210 (0.196, 0.226)	--^e^	0.054 (0.050–0.058)	0.049 (0.044, 0.054)	0.005 (−0.002, 0.011)
Eye	--^d^	--^d^	–	0.081 (0.074, 0.088)	0.073 (0.063, 0.083)	0.008 (−0.004, 0.019)	0.029 (0.027, 0.032)	0.029 (0.026, 0.032)	--^e^
Ear	--^d^	--^d^	–	0.017 (0.014, 0.020)	0.017 (0.011, 0.020)	--^e^	0.016 (0.014, 0.018)	0.012 (0.010, 0.015)	0.004 (−0.0001, 0.006)
Skin	0.087 (0.070, 0.102)	0.095 (0.084, 0.107)	--^e^	0.083 (0.075, 0.090)	0.093 (0.082, 0.104)	--^e^	0.029 (0.027, 0.032)	0.023 (0.020, 0.027)	0.006 (0.002, 0.010)

^a^ Comparing those who fish to the unexposed group (non-water recreators) in CHEERS

^b^ Comparing those who engage in incidental-contact water recreation (kayaking, canoeing, boating, rowing) to unexposed group (non-water recreators) in CHEERS

^c^ Comparing those who have any contact with beach water (swimmers/waders) to the unexposed group (non-water recreators) in NEEAR

^d^ Anglers not expected to have excess risk of respiratory, eye, ear, or skin illness due to nature of fishing exposure

^e^ No AR calculated, since no excess of illness among the exposed compared to unexposed

**Table 3:** Estimated annual number of cases of mild and moderate illness (C90) due to water recreation, by activity, in millions ^a^

Activity	AGI	Respiratory	Eye	Ear	Skin
Swimming	29.14 (23.09–34.58)	9.22 (1.29–16.66)	--^b^	6.87 (2.62–10.25)	12.06 (6.99–16.87)
Kayaking	0.54 (0.11–0.97)	--^b^	0.63 (0.04–1.29)	--^b^	--^b^
Rowing	0.38 (0.03–0.76)	--^b^	0.42 (0.03–0.85)	--^b^	--^b^
Canoeing	0.78 (0.15–1.38)	--^b^	0.91 (0.05–1.84)	--^b^	--^b^
Motor boating	4.77 (0.94–8.50)	--^b^	5.59 (0.36–11.31)	--^b^	--^b^
Fishing	15.27 (4.12–25.12)	--^b^	--^b^	--^b^	--^b^
Total	50.95 (36.59–63.42)	9.22 (1.29–16.66)	7.55 (0.49–15.28)	6.87 (2.62–10.25)	12.06 (6.99–16.87)
Moderate Severity^c^	7.2%	13.7%	10.4%	24.3%	5.4%

^a^ Calculated based on estimated number of water recreation events and the attributable risk of illness, may not sum to estimated totals due to rounding

^b^ No excess illnesses expected, based on the attributable risk

^c^ Percent with moderate severity illness (those who contact their HCP or go to an ED)

Based on the distribution of illness severity in NEEAR and CHEERS, the number of cases of illness, and the cost of each severity category of illness, we estimate an annual economic burden of recreational waterborne illness of $2.9 (C90: 2.2, 3.7) billion (Table 4). Lost productivity accounted for $663 million (23% of the total), due to the large number of individuals who could not attend work. Moderate severity illnesses account for a small percent of waterborne illness (9.2%), yet accounted for the majority of the economic burden (C90: $1.9 billion, or 65% of the total). Among those with moderate illness, HCP and ED visits accounted for 81% of the economic cost. While death was rare, the costs of lost life accounted for 97% of the estimated $233.7 (C90: 108.5, 613.9) million attributed to severe illness events. The cost of illness per case of illness ranged from $9.50 (C90: 8.62–10.34) for with mild illness, $233.38 (C90: 202.30–277.15) for moderate illness, and $302,685 (C90: 156,803–775,605) for the most severe illnesses. The complete breakdown of economic burden by illness type can be found in the (Additional file 1: Tables S11-S13).

**Table 4:** Total and per-case cost estimates (in millions of US dollars) of mild, moderate, and severe waterborne illnesses; mean (C90) ^a^

		Mild	Moderate	Severe	
Total cost in Millions of US Dollars	OTC medication	242.2 (182.0–295.1)	33.5 (22.7–42.4)	0.004 (0.001–0.009)	
	Prescription medication	53.1 (26.3–67.5)	126.0 (82.5–160.0	0.03 (0.01–0.08)	
	Doctor office visit	–	615.0 (427.3–803.3)	0.16 (0.06–0.36)	
	ED	–	924.9 (631.0–1226.7)	0.22 (0.09–0.50)	
	Hospitalization	–	–	6.79 (3.19–14.07)	
	Death	–	–	225.79 (103.55–599.07)	
	Sequelae	**–**	**–**	0.38 (0.03–1.44)	
	Lost Productivity	461.3 (342.2–586.5)	201.1 (146.2–286.5)	0.31 (0.13–0.69)	
	Total	756.7 (568.3–928.3)	1900.5 (1393.6–2400.6)	233.68 (108.49–613.95)	
	*Cost per case* *in US Dollars*	*9.50* *(8.62–10.34)*	*233.38* *(202.30–277.15)*	*302,685.57* *(156,803.58–775,605.39)*	
		*Total cost of Recreational Waterborne Illness in Millions*			2890.9 (2206.0–3663.7)

^a^ Estimated costs in 2007 dollars, may not sum to estimated totals due to rounding

## Discussion

We estimate that swimming/wading, paddling, motor-boating, and fishing in US surface waters account for over 90 million cases of GI, respiratory, ear, eye, or skin-related illnesses with a wide range of severity. The estimated annual economic burden of recreational waterborne illnesses is approximately $2.9 (C90: 2.2, 3.7) billion. While mild illness was common, approximately 65% of the cost was attributable to illness that resulted in contact with an HCP or evaluation in an ED.

Dwight et al. [9] estimated the annual economic burden (direct and indirect costs) due to water recreation at two beaches in Orange County, California to be $3.3 million in 2001 dollars ($3.9 million in 2007 dollars) based on lost productivity and healthcare office visit costs. In the current analysis, we estimated 1.9 billion swimming events occur annually in the US (Table 1). An estimated 5.5 million swimming events occur annually at Orange County beaches [26], or approximately 0.3% of the swimming events in the US. If costs estimated by Dwight and colleagues were scaled up, the national estimate of the annual economic burden due to swimming in surface water would be more than $1 billion. In contrast to the current study, Dwight et al. did not include costs associated with hospitalization, deaths, or medications, and was focused on adults 18 years of age and older, yet their estimate is still within the margin of error for our estimate ($1.7 (C90: 1.4, 2.0) billion) among swimmers/waders. Both cohort studies in the current analysis included children, which could also account for the cost differential between our analysis and the one conducted by Dwight and colleagues, especially since children (aged 0–10) appear to experience the majority of moderate illnesses (Additional file 1: Table S9).

Another study by Given et al. (2006) examined the number of excess gastrointestinal illnesses due to swimming at 28 beaches in southern California [27]. This study estimated that 627,800 and 1,479,200 excess gastrointestinal illnesses occur annually due to swimming in Los Angeles and Orange County California, or between 2 and 5% of our national estimate. Using the per-case cost estimate from Dwight et al. [9], costs (time missed from work, doctor visits) Given et al. (2006) estimated costs of GI illness to range from $21 to $51 million in 2000 dollars, or about 1–3% of our national estimate of costs attributable to gastrointestinal illness. Therefore, costs estimated by Given et al. (2006) are consistent with our national estimate, despite not including information regarding hospitalization or medication use.

Collier et al. [10] estimated that US hospitalization costs for illnesses caused by microbes primarily transmitted by water (giardiasis, cryptosporidiosis, Legionnaires’ disease, otitis externa, and non-tuberculous mycobacterial infection) were $970 million annually and illnesses only partially transmitted by water (campylobacteriosis, salmonellosis, shigellosis, hemolytic-uremic syndrome, and toxoplasmosis) accounted for $860 million in hospitalizations annually. In that study, researchers estimated the annual number of infections caused by specific pathogens, then estimated the proportion possibly due to water exposure (not specifically surface water recreation). In the present study, we estimated costs due to hospitalizations from recreation in untreated surface water to be $6.8 (C90: 3.2 to $14.1) million, or approximately 4% of the Collier et al. estimate. This discrepancy between the two estimates could be largely due to their inclusion of illnesses that may have been acquired via drinking water, swimming pools, and water parks, in addition to surface water recreation, or not via water exposure. From 2007 to 2008, approximately 90% of all outbreaks and 95% of all outbreak cases were associated with treated recreational water, such as swimming pools, spas, and water parks [28]. Also, pathogens responsible for sporadic illness are rarely identified [29]. Thus, estimation of costs of sporadic illness that rely upon identifying specific pathogens in stool samples of ill recreators will underestimate the costs overall in the absence of multipliers that account for underdiagnosis and underreporting.

Foodborne illness has been estimated to cost between $51–77 billion each year, depending on assumptions regarding lost productivity and pain and suffering [19]. Cost of illness due to surface water recreation would be expected to be a fraction of the costs of foodborne illnesses, as food consumption is a daily occurrence, unlike water recreation.

A strength of the present study is large prospective cohort studies containing a combined over 35,000 participants were used to estimate risk of illness attributable to surface water recreation. These studies obtained data directly from those engaged in water recreation regarding illness, healthcare utilization, and lost productivity. Prior studies of the cost of waterborne illness that utilized healthcare claims and hospital discharge data [10] but had no available information regarding water exposure. The present study used a combination of cohort and outbreak data with known water exposure, and other components of economic burden, such as costs associated with lost productivity and medication use. Unlike the prior estimate of the cost of waterborne illness at a California beach [9], the present study is likely more representative of surface water recreation nationally, as it included data from freshwater and marine beaches, as well as urban inland waters. Furthermore, we incorporated additional costs components in the estimate (hospitalizations, sequelae, and mortality).

Our findings are subject to several limitations. The accuracy of the economic burden estimate depends on the accuracy of the estimation of the total number of people in the US engaging in water recreation and an estimate of the total number of person-days associated with a particular water activity. We only used estimates of the number of warmwater fishing events, (fishing in rivers, streams, lakes, and oceans); less common types of fishing, including andromodous, coldwater, and saltwater, [1], were not considered. We also focused primarily on five major health outcomes (AGI, respiratory, ear, eye, and skin symptoms) in addition to severe pathogen-specific illnesses. Harmful algal blooms (HABs) produce toxins that can result in AGI, respiratory, skin, eye, ear, and neurological symptoms [30], and have been estimated to be responsible for $0.5–$4 million in respiratory ED visits in Sarasota Country, Florida annually [31]. Exposures to HABs may have a significant economic impact, but were not included in our analysis. We considered sequelae (hemolytic-uremic syndrome with or without end stage renal disese, reactive arthritis, and Guillain-Barré syndrome) as a result of illness among those who were hospitalized. However, we did not consider costs of reactive arthritis, which can be treated in an outpatient setting, for any individuals who were not hospitalized, which could have also underestimated costs among those with moderate illness.

In addition, our analysis assessed risks for individual illnesses, but it is possible that water recreation can result in the development of more than one type of illness (ie AGI and respiratory illness). While this would likely not impact the attributable fraction, it could be expected to have an impact on cost. For example, a person experiencing both AGI and respiratory symptoms who saw a HCP or visited an ED, most likely would have been treated for both symptoms concurrently, opposed to being charged for two individual visits. This could have resulted in an overestimation of economic burden. Additionally, we were not able to differentiate individuals whose symptoms were due to infection versus other causes; likewise, our reliance on symptom reporting means that we were not able to estimate the number of asymptomatic infections.

In our analysis, we did not estimate economic burden according to age category. Since children are more likely to have contact with surface water [32] and are at an increased risk of illness [33], we would expect that some of the economic burden for water recreation to be among children. We found that according epidemiology studies, a large portion of mild illness is among adults aged 20–54, while the majority of moderate illness is among children aged 0–10 (Additional file 1: Table S9). While we expect risk to differ by age, whether the costs differ according to age is less clear. With regard to lost productivity for mild and moderate illnesses, we used an estimate of the mean (and the 5th and 95th percentile of the distribution) estimate of time missed from work estimated from epidemiology studies, which already takes into account the distribution of the age of those who become ill. However, we know less about the age distribution among those who develop severe (hospitalizations/death) illnesses following water recreation.

CHEERS and NEEAR were conducted at study locations that were likely impacted by human fecal pollution (particularly CHEERS [34]). Thus, the attributable risks calculated within the context of these studies may have potentially overestimated the risk of recreational waterborne illnesses relative to surface water recreation overall. For example, illness rates at Boqueròn beach in Puerto Rico, had lower concentrations of fecal indicators and observed illness rates were much lower than those in other NEEAR sites [35, 36], However, Boquerón is atypical of US beaches as it is tropical and symptoms following water recreation at Boquerón beach have been strongly linked to cyanobacteria [37], as opposed to fecal indicators. Water quality at NEEAR beaches generally met local water quality criteria and health risks observed there should reflect those at comparable beaches elsewhere. The range of water quality observed at waters where recreation took place in the epidemiologic studies is well within those described in a national summary of water quality [38]. It is reasonable to assume that as water quality improves, economic burden would decrease, yet further study is necessary to make this conclusion.

In addition, since the attributable risks were based on epidemiologic studies, there is a possibility of over-reporting of illness if they had at baseline concerns about health risks due to water recreation, a phenomenon referred to as risk perception bias [39]. The use of the distributions of the attributable risks may allow for some degree of heterogeneity among beachgoers. Some attributable risks confidence intervals estimated for specific outcomes include zero (Table 2). To be conservative, we included these negative values at the lower bound in our Monte Carlo simulation. When we truncated risk at zero, we did not observe significant differences in the mean costs or in the C90.

Our cost estimates were approximated using 2007 USD. We chose to estimate the economic burden based on the year 2007 since it was a year in which several of our data sources overlapped. Accounting for inflation or 2007 USD estimate of $2.9 (2.2, 3.7) billion would be approximately $3.4 (2.6, 4.4) billion in 2017 USD. However, since there have been several changes to the healthcare system in recent years, it is inappropriate to express our estimate in more recent dollars.

The use of outbreak and passive surveillance multipliers based on foodborne illness data may not be ideal for adjusting for undercounting of recreational waterborne outbreaks. Currently, extensive surveillance data exists for foodborne illnesses, which were used to construct the multipliers [14]. Active surveillance systems for recreational waterborne illnesses do not exist in the US and it is unknown whether outbreak and passive surveillance multipliers are appropriate for recreational waterborne illness. However, these multipliers, based on several ratios of laboratory-confirmed cases of foodborne illness to cases identified through foodborne outbreak surveillance, are currently the best available.

## Conclusions

The annual economic burden due to surface water recreation ranges between $2.2–3.7 billion. Estimates of costs of prevention activities (beach monitoring and notification programs), and reduction of wastewater and storm-water discharges near beaches, should be viewed in context of this burden estimate. To date, approximately $140 million has been allocated toward beach-water protection programs since 2001 [40] or about $10 million annually, a small fraction of annual estimated illness burden. Efforts to reduce severity of illness among water recreators should be explored to reduce total economic burden and encourage more individuals to enjoy surface water recreation. Furthermore, we hope that future investigations build upon our work by estimating the health care costs saved as a function of improvements in water quality.

## Additional file

Additional file 1: Table S1. Data sources and assumptions utilized in calculating the economic burden of recreational waterborne illness. **Table S2.** Covariates used in logistic regression model to estimate attributable risk. **Table S3.** ICD-9-CM codes to determine ED and hospital costs. **Table S4.** Parameters used to estimate the number of mild and moderate illnesses. **Table S5.** Parameters used to estimate the number of severe illnesses. **Table S6.** Parameters used to estimate the cost of mild illness. **Table S7.** Parameters used to estimate the cost of moderate illness. **Table S8.** Parameters used to estimate the cost of severe illness. **Table S9.** Proportion of mild and moderate illnesses by age category. **Table S10.** Estimated number of outbreak cases, hospitalizations, and deaths due to water recreation (C90). **Table S11.** Total cost of mild waterborne illness; mean (C90). **Table S12.** Total cost of moderate waterborne illness; mean (C90). **Table S13.** Total cost of severe waterborne illness; mean (C90). (DOCX 147 kb)

## Abbreviations

AGI: Acute gastrointestinal illness
AHRQ: Agency for Healthcare Research and Quality
C90: Central 90% of values
CHEERS: Chicago Health, and Environmental Exposure, and Recreation Study
ED: Emergency department
HAB: Harmful algal bloom
HCP: Health care provider
NEDS: Nationwide Emergency Department Sample
NEEAR: National Environmental and Epidemiological Assessment of Recreational Water
NIS: Nationwide Inpatient Sample
NSRE: National Survey on Recreation and Environment
OTC: Over-the-counter
VSL: Value of a statistical life
WBDOSS: Waterborne Disease and Outbreak Surveillance System
