# Supplementary material for: Estimate of incidence and cost of recreational waterborne illness on United States surface waters
Source: Environ Health. 2018 Jan 9;17:3. doi: 10.1186/s12940-017-0347-9 (PMC5759255; doi:10.1186/s12940-017-0347-9)
Supplement: Additional file 1: Table S1. — Data sources and assumptions utilized in calculating the economic burden of recreational waterborne illness. Table S2. Covariates used in logistic regression model to estimate attributable risk. Table S3. ICD-9-CM codes to determine ED and hospital costs. Table S4. Parameters used to estimate the number of mild and moderate illnesses. Table S5. Parameters used to estimate the number of severe illnesses. Table S6. Parameters used to estimate the cost of mild illness. Table S7. Parameters used to estimate the cost of moderate illness. Table S8. Parameters used to estimate the cost of severe illness. Table S9. Proportion of mild and moderate illnesses by age category. Table S10. Estimated number of outbreak cases, hospitalizations, and deaths due to water recreation (C90). Table S11. Total cost of mild waterborne illness; mean (C90). Table S12. Total cost of moderate waterborne illness; mean (C90). Table S13. Total cost of severe waterborne illness; mean (C90). (DOCX 147 kb) [file 12940_2017_347_MOESM1_ESM.docx]

**Title**: Burden of Recreational Waterborne Illness at United States Surface Waters

**Table of Contents**

|  | **Page** |
| --- | --- |
| Table S1: Data sources and assumptions utilized in calculating the economic burden of recreational waterborne illness | 2 |
| Data Sources: Illness Occurrence | 3-4 |
| Table S2: Covariates used in logistic regression model to estimate attributable risk | 5 |
| Table S3: ICD-9-CM codes to determine ED and hospital costs | 6 |
| Table S4: Parameters used to estimate the number of mild and moderate illnesses | 7 |
| Table S5: Parameters used to estimate the number of severe illnesses | 8 |
| Table S6: Parameters used to estimate the cost of mild illness | 9 |
| Table S7: Parameters used to estimate the cost of moderate illness | 10 |
| Table S8: Parameters used to estimate the cost of severe illness | 11 |
| Table S9: Proportion of mild and moderate illnesses by age category | 13 |
| Table S10: Estimated number of outbreak cases, hospitalizations, and deaths due to water recreation (C90) | 14 |
| Table S11: Total cost of mild waterborne illness; mean (C90) | 15 |
| Table S12: Total cost of moderate waterborne illness; mean (C90) | 16 |
| Table S13: Total cost of severe waterborne illness; mean (C90) | 17 |
| References | 18-20 |

Table S1: Data sources and assumptions utilized in calculating the economic burden of recreational waterborne illness

| **Cost Component** |  | **Source of Data** | **Assumptions** |
| --- | --- | --- | --- |
| **Medications** | Price | (NEEAR; (Frenzen 2007; Scharff 2011) | Costs of medications for mild and moderate illnesses reported in NEEAR representative of US population. Cost of $44.67 for prescription medications for all cases of severe illness. |
|  | Quantity | NEEAR | Proportion taking medications in NEEAR is representative of the US population. |
| **HCP** | Price | (PMIC 2007) | Moderate ear, eye, and skin illness equivalent to E/M level-2, GI and respiratory illnesses equivalent to E/M level-3. Severe illnesses equivalent to E/M level-4 |
|  | Quantity | (NEEAR; (ERS 2014) | Proportion visiting an in NEEAR is representative of the US population. Cases of severe foodborne illness are similar to cases of with illness recreational waterborne illness. |
| **ED** | Price | (NEDS; (ERS 2014) | Moderate ear, eye, and skin illness equivalent to E/M level-2, GI and respiratory illnesses equivalent to E/M level-3.  ED cost of Bacterial Meningitis (ICD-9-CM: 320.0) reflective of ED costs related to PAM.  ED costs aggregated for foodborne *Vibrio* infections reflective of ED costs for waterborne *Vibrio* infections. |
|  | Quantity | (NEEAR; (ERS 2014) | Proportion visiting an ED in NEEAR is representative of the US population. Cases of severe foodborne illness are similar to cases of with illness recreational waterborne illness. |
| **Missed days of work or daily activity** | Price | (US Census) | Daily wage calculated from median income for the US population representative of daily wage for water recreators. |
|  | Quantity | (NEEAR; (ERS 2014) | Proportion missing work in NEEAR is representative of the US population. |
| **Hospital Admission** | Price | (NIS) | Hospitalization cost of Bacterial Meningitis (ICD-9-CM: 320.0) reflective of hospitalization costs related to PAM. Hospitalization costs for foodborne *Vibrio* infections are reflective of hospitalization costs for waterborne *Vibrio* infections. |
|  | Quantity | (Dechet et al. 2008; Dziuban et al. 2006; Goarant et al. 2009; Hlavsa et al. 2011; Hlavsa et al. 2014; Mead et al. 1999; Pond 2005; Scallan et al. 2011; Yoder et al. 2008; Yoder et al. 2010) | Wide range of estimated proportion of pathogen-specific illnesses that result in hospitalization. |
| Sequele | Price | (Frenzen et al. 2005; Frenzen 2008; Glennas et al. 1994; Scharff 2011; Townes et al. 2008) | Average costs of sequelae were estimated for Guillain-Barré syndrome ($344.39/ *Campylobacter* hospitalization), hemolytic-uremic syndrome ($2,359.90/ *E. coli* 0157H7 hospitalization) with or without end stage renal disease, and reactive arthritis (ReA) ($52.94/ *Campylobacter* and *Shigella* hospitalizations) |
|  | Quantity | (Dechet et al. 2008; Dziuban et al. 2006; Goarant et al. 2009; Hlavsa et al. 2011; Hlavsa et al. 2014; Mead et al. 1999; Pond 2005; Scallan et al. 2011; Yoder et al. 2008; Yoder et al. 2010) |  |
| Death | Price | (US EPA 2010) | Age-invariant estimate |
|  | Quantity | (Dechet et al. 2008; Dziuban et al. 2006; Goarant et al. 2009; Hlavsa et al. 2011; Hlavsa et al. 2014; Mead et al. 1999; Pond 2005; Scallan et al. 2011; Yoder et al. 2008; Yoder et al. 2010) | Estimates from outbreaks and the literature |

## *Data sources: Illness occurrence*

Cohort studies designed to evaluate the short-term health risks of water recreation were used to estimate the incidence of illness. Data from the National Epidemiological and Environmental Assessment of Recreational Water (NEEAR) were used to estimate the incidence of illness attributable to swimming and wading (Dorevitch et al. 2012a; Dorevitch et al. 2015; Wade et al. 2006; Wade et al. 2008; Wade et al. 2010a). Data from the Chicago Health Environmental Exposure and Recreation Study (CHEERS), were used to estimate the incidence of illness attributable to incidental-contact recreation (kayaking, canoeing, rowing, motor boating, and fishing) (Dorevitch et al. 2012a; Dorevitch et al. 2015). Both studies were similar in design and have been summarized previously(Dorevitch et al. 2012a; Dorevitch et al. 2015; Wade et al. 2006; Wade et al. 2008; Wade et al. 2010a). While these studies describe the incidence rate of illness among water recreators, not all cases of illness among water recreation are due to water recreation. Attributable risk which takes into account differences in risk between water recreators and non-water recreators, were expressed as the number of illnesses attributable to water recreation per 1,000 recreators. These were calculated for GI, respiratory, ear, eye, and skin symptoms separately for swimmers/waders, anglers, and all other incidental-contact recreators. Higher rates of illness have been observed among anglers (Dorevitch et al 2015), possibly due to water exposure and the handling of bait/fish (Roberts et al. 2007). Attributable risks were estimated from a logistic regression model of the relationship between any water exposure and each health endpoint, according to Equation 1, and have been described previously (DeFlorio-Barker et al. 2016b). Covariates used in the logistic models were determined *a priori* based on the relationships between any water exposure and each health endpoint (Supplement, Table S1). Analyses for assessing the AR’s were conducted using Stata 11 (StataCorp LP, College Station, Texas).

**Equation 1:**

$$AR=p_{1}-p_{0}$$

*Where* $p_{1}$*= probability of illness in the exposed,*

*and* $p_{0}$*=probability of illness in the unexposed*

The cohort studies provide vital information about the occurrence of sporadic cases of illness. Severe illness resulting in hospitalization was rare in the cohort studies, while WBDOSS has identified thousands of outbreak-associated cases of illness, many of which are severe enough to result in hospitalization. Thus the epidemiologic studies appear to identify relatively mild cases of illness while outbreak reports generally capture cases of much greater severity. For that reason we partitioned cases into three main categories: mild, moderate, and severe. Mild illnesses were defined as cases of illness not resulting in contact with a healthcare provider (HCP), moderate illnesses were defined as contact with a HCP either in a physician’s office or in the ED, while severe cases were defined as those requiring hospitalization (Hoffmann et al. 2012).

Table S2: Covariates used in logistic regression model to estimate attributable risk

|  | GI | Respiratory | Ear | Eye | Skin |
| --- | --- | --- | --- | --- | --- |
| *Demographics* |  |  |  |  |  |
| Age | IC,S/W | IC,S/W | IC,S/W | IC,S/W | IC,S/W |
| Gender | IC,S/W | IC,S/W | IC,S/W | IC,S/W | IC,S/W |
| Race | IC,S/W | IC,S/W | IC,S/W | IC,S/W | IC,S/W |
| *Underlying illness* |  |  |  |  |  |
| Chronic GI Condition | IC,S/W | IC,S/W |  |  |  |
| Preexisting respiratory condition |  | IC,S/W | IC,S/W |  |  |
| Preexisting diabetes | IC | IC | IC | IC | IC |
| Average daily bowel movements | IC |  |  |  |  |
| *Infection/immunity* |  |  |  |  |  |
| Prone to infection | IC | IC | IC | IC | IC |
| Antibiotic use in past 7 days | IC | IC |  |  |  |
| Recent antacid use | IC | IC |  |  |  |
| *Exposures unrelated to water recreation* |  |  |  |  |  |
| Recent contact with dog or cat | IC,S/W | IC,S/W |  |  | IC,S/W |
| Recent contact with other animal | IC,S/W | IC,S/W |  |  | IC,S/W |
| Recently ate raw fish or shell fish | IC,S/W |  |  |  | IC,S/W |
| Recently ate raw meat | IC,S/W |  |  |  |  |
| Recently ate hamburger | IC |  |  |  |  |
| Recently ate raw/runny eggs | IC,S/W |  |  |  |  |
| Recently ate fresh produce | IC |  |  |  |  |
| Recently ate packaged sandwich | IC |  |  |  |  |
| Recent contact to someone with GI symptoms | IC,S/W |  | IC,S/W | IC,S/W |  |
| Recent contact to someone with respiratory symptoms |  | IC |  | IC |  |
| Recent contact to someone with eye symptoms |  |  |  | IC |  |
| *Related to recreational activity* |  |  |  |  |  |
| Washing hands before eating/drinking after water recreation | IC,S/W | IC,S/W |  |  |  |
| Frequency of recreation at location of enrollment (0-365)  0-4 (referent group)  5-10 times  ≥11 times | IC,S/W | IC,S/W | IC,S/W | IC,S/W | IC,S/W |
| Recreation during follow-up | IC,S/W | IC,S/W | IC,S/W | IC,S/W | IC,S/W |
| Dug into the sand | S/W | S/W | S/W | S/W | S/W |
| Wore ear plugs |  |  | S/W |  |  |

IC=Incidental-contact, S/W=swimming/wading

Covariates chosen *a priori* and on available data in the two epidemiology studies

Table S3: ICD-9-CM codes to determine ED and hospital costs^a^

| **Symptom** | **ICD-9-CM** | **ICD-9-CM Description** |
| --- | --- | --- |
| **GI** | 009 | Infectious colitis, enteritis, and gastroenteritis |
|  | 008.8 | Viral gastroenteritis |
| **Ear** | 380.1 | Acute otitis externa |
|  | 380.12 | Acute swimmers' ear |
|  | 380.14 | Malignant otitis externa |
| **Eye** | 379.93 | Redness or discharge of eye |
|  | V74.4 | Bacterial conjunctivitis |
|  | 372.0 | Acute conjunctivitis |
|  | 372.00 | Acute conjunctivitis, unspecified |
| **Respiratory** | 465 | Acute upper respiratory infections of multiple or unspecified sites |
|  | 465.8 | Acute upper respiratory infections of other multiple sites |
|  | 465.9 | Acute upper respiratory infections of unspecified site |
|  | 786.2 | Cough |
|  | 460 | Acute nasopharyngitis (common cold) |
|  | 462 | Acute pharyngitis |
| **Skin** | 782.1 | Rash and other nonspecific skin eruption |
|  | 692.9 | Dermatitis |
| **Pathogens** | 004 | Shigellosis |
|  | 007.1 | Giardiasis |
|  | 007.4 | Cryptosporidiosis |
|  | 008 | Intestinal infection due to *E. coli* |
|  | 008.43 | Campylobacter |
|  | 008.63 | Norwalk virus |
|  | 100 | Leptospirosis |
|  | 136.2 ^b^ | Specific infections by free-living amebae; Meningoencephalitis due to *Naegleria* |

^a^*Vibrio* spp. cell sizes were too small (<10) to estimate costs from NEDS and NIS, used costs reported by EDS for *Vibrio*

^b^Not included in 2007 NEDS or NIS, Bacterial Meningitis (ICD-9-CM 320.0) used as a surrogate

Table S4: Parameters used to estimate the number of mild and moderate illnesses

| **Pathogen and Model Input** | **Distribution** | **Parameters** | **References** |
| --- | --- | --- | --- |
| **Proportion of US population 16 and over participating** |  |  |  |
| Motor boat | Beta-Pert (min, mode, max) | 0.229, 0.234, 0.239 | National Survey on Recreation and the Environment (Cordell 2012) |
| Kayak | Beta-Pert (min, mode, max) | 0.058, 0.060, 0.063 |  |
| Canoe | Beta-Pert (min, mode, max) | 0.094, 0.097, 0.100 |  |
| Row | Beta-Pert (min, mode, max) | 0.038, 0.040, 0.042 |  |
| Fish | Beta-Pert (min, mode, max) | 0.232, 0.237, 0.239 |  |
| Swimming/wading | Beta-Pert (min, mode, max) | 0.409, 0.415, 0.420 |  |
| **Attributable Fractions, by illness^a^** |  |  |  |
| GI |  |  | Swimming/wading estimates derived from NEEAR study (Wade et al. 2006; Wade et al. 2008; Wade et al. 2010).  Incidental contact estimates derived from CHEERS study (Dorevitch et al. 2012a). |
| Swimming/wading | Beta-Pert (min, mode, max) | 0.010, 0.015, 0.020 |  |
| Incidental contact, no fishing | Beta-Pert (min, mode, max) | -0.002, 0.006, 0.014 |  |
| Incidental contact, fishing | Beta-Pert (min, mode, max) | -0.003, 0.015, 0.030 |  |
| Respiratory |  |  |  |
| Swimming/wading | Beta-Pert (min, mode, max) | -0.002, 0.005, 0.011 |  |
| Eye |  |  |  |
| Incidental contact, no fishing | Beta-Pert (min, mode, max) | -0.004, 0.008, 0.020 |  |
| Ear |  |  |  |
| Swimming/wading | Beta-Pert (min, mode, max) | 0.000, 0.004, 0.006 |  |
| Skin |  |  |  |
| Swimming/wading | Beta-Pert (min, mode, max) | 0.002, 0.006, 0.010 |  |
| **Moderate illness assumptions^b^** |  |  |  |
| GI illnesses that are moderate | Beta-Pert (min, mode, max) | 0.059, 0.073, 0.086 | Estimates derived from NEEAR study (Wade et al. 2006; Wade et al. 2008; Wade et al. 2010). |
| Respiratory illnesses that are moderate | Beta-Pert (min, mode, max) | 0.115, 0.137, 0.159 |  |
| Eye illnesses that are moderate | Beta-Pert (min, mode, max) | 0.077, 0.104, 0.131 |  |
| Ear illnesses that are moderate | Beta-Pert (min, mode, max) | 0.193, 0.243, 0.293 |  |
| Skin illnesses that are moderate | Beta-Pert (min, mode, max) | 0.035, 0.054, 0.073 |  |

^a^ Multiplied by total number of recreators in each category to yield total number of illnesses in each category

^b^ Multiplied by total number of illnesses to yield total number of moderate illnesses (total illnesses-total number of moderate illnesses = total number of mild illnesses)

Table S5: Parameters used to estimate the number of severe illnesses

| **Pathogen and Model Input** | **Distribution** | **Parameters** |  |
| --- | --- | --- | --- |
| ***Campylobacter*** |  |  |  |
| Number in outbreaks | Lognormal, mean (sd) | 130.05 (360.7) | WBDOSS* |
| Proportion hospitalized | Beta-Pert (min, mode, max) | 0.17, 0.13, 0.25 | (Mead et al. 1999; Pond 2005; Scallan et al. 2011) |
| Proportion dead | Beta-Pert (min, mode, max) | 0, 0.001, 0.004 | (Pond 2005; Scallan et al. 2011) |
| ***Cryptosporidium*** |  |  |  |
| Number in outbreaks | Lognormal, mean (sd) | 191.25 (221.65) | WBDOSS* |
| Proportion hospitalized | Beta-Pert (min, mode, max) | 0.13, 0.15, 0.25 | (Pond 2005; Scallan et al. 2011) |
| Proportion dead | Beta-Pert (min, mode, max) | 0, 0.0001, 0.003 | (Pond 2005; Scallan et al. 2011) |
| ***E. coli 0157H7*** |  |  |  |
| Number in outbreaks | Lognormal, mean (sd) | 252.45 (536.97) | WBDOSS* |
| Proportion hospitalized | Beta-Pert (min, mode, max) | 0.29, 0.3,0.46 | (Pond 2005) |
| Proportion dead | Uniform (min, max) | 0.005, 0.008 | (Mead et al. 1999; Pond 2005; Scallan et al. 2011) |
| ***Giardia*** |  |  |  |
| Number in outbreaks | Lognormal, mean (sd) | 28.05 (72.57) | WBDOSS* |
| Proportion hospitalized | Beta-Pert (min, mode, max) | 0, 0.09, 0.13 | (Pond 2005; Scallan et al. 2011) |
| Proportion dead | Uniform (min, max) | 0, 0.001 | (Pond 2005) |
| ***Leptospira*** |  |  |  |
| Number in outbreaks | Lognormal, mean (sd) | 124.95 (369.03) | WBDOSS* |
| Proportion hospitalized | Beta-Pert (min, mode, max) | 0.3, 0.5, 0.652 | (Goarant et al. 2009; Pond 2005) |
| Proportion dead | Beta-Pert (min, mode, max) | 0.01, 0.035, 0.14 | (Goarant et al. 2009; Pond 2005) |
| ***Naegleria fowleri*** |  |  |  |
| Number in outbreaks | Triangular (min, mode, max) | 0, 4, 8 | WBDOSS* |
| Proportion hospitalized | Point Estimate | 1.0 | (Yoder et al. 2010) |
| Proportion dead | Uniform (min, max) | 0.99, 1.0 | (Yoder et al. 2010) |
| ***Norovirus*** |  |  |  |
| Number in outbreaks | Lognormal, mean (sd) | 879.75 (744.73) | WBDOSS* |
| Proportion hospitalized | Point Estimate | 0.03 | WBDOSS* |
| Proportion dead | Point Estimate | 0.001 | (Scallan et al. 2011) |
| ***Shigella*** |  |  |  |
| Number in outbreaks | Lognormal, mean (sd) | 627.3 (802.81) | WBDOSS* |
| Proportion hospitalized | Beta-Pert (min, mode, max) | 0.139, 0.2, 0.22 | (Pond 2005; Scallan et al. 2011) |
| Proportion dead | Beta-Pert (min, mode, max) | 0.001, 0.002, 0.002 | (Pond 2005; Scallan et al. 2011) |
| ***Vibrio parahaemolyticus*** |  |  |  |
| Number in outbreaks | Lognormal, mean (sd) | 21.63 (5.63) | WBDOSS*; (CDC 2012) |
| Proportion hospitalized | Beta-Pert (min, mode, max) | 0.33, 0.39, 0.5 | WBDOSS*; (CDC 2012) |
| Proportion dead | Beta-Pert (min, mode, max) | 0, 0.008, 0.048 | WBDOSS*; (CDC 2012) |
| ***Vibrio ahaemolyticus*** |  |  |  |
| Number in outbreaks | Lognormal, mean (sd) | 38.32 (16.38) | WBDOSS*; (CDC 2012) |
| Proportion hospitalized | Beta-Pert (min, mode, max) | 0.034, 0.14, 0.21 | WBDOSS*; (CDC 2012);(Dechet et al. 2008) |
| Proportion dead | Beta-Pert (min, mode, max) | 0, 0.013, 0.042 | WBDOSS*; (CDC 2012); (Scallan et al. 2011) |
| ***Vibrio* spp., other** |  |  |  |
| Number in outbreaks | Lognormal, mean (sd) | 7.52 (2.25) | WBDOSS*; (CDC 2012) |
| Proportion hospitalized | Beta-Pert (min, mode, max) | 0.11, 0.34, 0.56 | WBDOSS*; (CDC 2012) |
| Proportion dead | Beta-Pert (min, mode, max) | 0, 0.052, 0.2 | WBDOSS*; (CDC 2012) |
| ***Vibrio vulnificus*** |  |  |  |
| Number in outbreaks | Lognormal, mean (sd) | 29.7 (12.98) | WBDOSS*; (CDC 2012) |
| Proportion hospitalized | Beta-Pert (min, mode, max) | 0.63, 0.79, 1.0 | WBDOSS*; (CDC 2012) |
| Proportion dead | Beta-Pert (min, modal, max) | 0.05, 0.16, 0.24 | WBDOSS*; (CDC 2012) |
| ***Vibrio cholera*** |  |  |  |
| Number in outbreaks | Lognormal, mean (sd) | 4.24 (1.73) | WBDOSS*; (CDC 2012) |
| Proportion hospitalized | Beta-Pert (min, mode, max) | 0, 0.32, 0.8 | WBDOSS*; (CDC 2012) |
| Proportion dead | Beta-Pert (min, mode, max) | 0, 0.06, 0.1 | WBDOSS*; (CDC 2012) |

*(Dziuban et al. 2006; Hlavsa et al. 2011; Hlavsa et al. 2014; Yoder et al. 2004; Yoder et al. 2008)

Table S6: Parameters used to estimate the cost of mild illness^a^

| **Pathogen and Model Input for Mild Illness** | **Distribution** | **Parameters** |
| --- | --- | --- |
| **GI illness** |  |  |
| Proportion that take OTC | Beta-Pert (min, mode, max) | 0.36, 0.39, 0.41 |
| OTC medication cost | Lognormal, mean (sd) | 7.20 (0.34) |
| Proportion that take prescription | Beta-Pert (min, mode, max) | 0.01, 0.01, 0.02 |
| Prescription medication cost | Lognormal, mean (sd) | 19.98 (3.52) |
| Proportion that miss work | Beta-Pert (min, mode, max) | 0.05, 0.06, 0.07 |
| Days of work missed | Triangular (min, mode, max) | 1.01, 1.04, 1.07 |
| **Respiratory illness** |  |  |
| Proportion that take OTC | Beta-Pert (min, mode, max) | 0.61, 0.65, 0.68 |
| OTC medication cost | Lognormal, mean (sd) | 7.42 (0.35) |
| Proportion that take prescription | Beta-Pert (min, mode, max) | 0.02, 0.04, 0.05 |
| Prescription medication cost | Lognormal, mean (sd) | 27.31 (8.31) |
| Proportion that miss work | Beta-Pert (min, mode, max) | 0.02, 0.04, 0.05 |
| Days of work missed | Triangular (min, mode, max) | 1.01, 1.34, 1.66 |
| **Eye illness** |  |  |
| Proportion that take OTC | Beta-Pert (min, mode, max) | 0.39, 0.44, 0.49 |
| OTC medication cost | Lognormal, mean (sd) | 7.39 (0.56) |
| Proportion that take prescription | Beta-Pert (min, mode, max) | 0.07, 0.10, 0.12 |
| Prescription medication cost | Lognormal, mean (sd) | 23.45 (5.37) |
| **Ear illness** |  |  |
| Proportion that take OTC | Beta-Pert (min, mode, max) | 0.30, 0.37, 0.43 |
| OTC medication cost | Lognormal, mean (sd) | 6.79 (0.88) |
| Proportion that take prescription | Beta-Pert (min, mode, max) | 0.02, 0.05, 0.07 |
| Prescription medication cost | Lognormal, mean (sd) | 17.79 (3.96) |
| Proportion that miss work | Beta-Pert (min, mode, max) | 0.01, 0.03, 0.06 |
| Days of work missed | Triangular (min, mode, max) | 0.45, 1.29, 2.12 |
| **Skin illness** |  |  |
| Proportion that take OTC | Beta-Pert (min, mode, max) | 0.47, 0.53, 0.58 |
| OTC medication cost | Lognormal, mean (sd) | 5.80 (0.27) |
| Proportion that take prescription | Beta-Pert (min, mode, max) | 0.02, 0.04, 0.06 |
| Prescription medication cost | Lognormal, mean (sd) | 24.90 (8.85) |

^a^derived from NEEAR study (Wade et al. 2006; Wade et al. 2008; Wade et al. 2010).

Table S7: Parameters used to estimate the cost of moderate illness

| **Pathogen and Model Input for Moderate Illness** | **Distribution** | **Parameters** |
| --- | --- | --- |
| **GI illness ^a^** |  |  |
| Proportion that go to doctor | Beta-Pert (min, mode, max) | 0.88, 0.93, 0.98 |
| Proportion that take OTC | Beta-Pert (min, mode, max) | 0.51, 0.61, 0.71 |
| OTC medication cost | Lognormal, mean (sd) | 8.32 (1.14) |
| Proportion that take prescription | Beta-Pert (min, mode, max) | 0.47, 0.57, 0.67 |
| Prescription medication cost | Lognormal, mean (sd) | 27.54 (5.97) |
| Proportion that go to the ED | Beta-Pert (min, mode, max) | 0.17, 0.26, 0.35 |
| ED visit charge^b^ | Normal, mean (sd)^b^ | 1,328.61 (46.52) |
| Proportion that miss work | Beta-Pert (min, mode, max) | 0.15, 0.23, 0.31 |
| Days of work missed | Triangular (min, mode, max) | 0.98, 1.28, 1.60 |
| **Respiratory illness ^a^** |  |  |
| Proportion that go to doctor | Beta-Pert (min, mode, max) | 0.95, 0.98, 1.00 |
| Proportion that take OTC | Beta-Pert (min, mode, max) | 0.55, 0.63, 0.72 |
| OTC medication cost | Lognormal, mean (sd) | 7.70 (0.81) |
| Proportion that take prescription | Beta-Pert (min, mode, max) | 0.58, 0.66, 0.75 |
| Prescription medication cost | Lognormal, mean (sd) | 22.22 (3.57) |
| Proportion that go to the ED | Beta-Pert (min, mode, max) | 0.06, 0.11, 0.17 |
| ED visit charge^b^ | Normal, mean (sd)^b^ | 617.00 (14.62) |
| Proportion that miss work | Beta-Pert (min, mode, max) | 0.10, 0.16, 0.23 |
| Days of work missed | Triangular (min, mode, max) | 0.99, 1.43, 1.88 |
| **Eye illness ^a^** |  |  |
| Proportion that go to doctor | Beta-Pert (min, mode, max) | 1.00, 1.00, 1.00 |
| Proportion that take OTC | Beta-Pert (min, mode, max) | 0.22, 0.36, 0.50 |
| OTC medication cost | Lognormal, mean (sd) | 9.67 (1.91) |
| Proportion that take prescription | Beta-Pert (min, mode, max) | 0.66, 0.78, 0.90 |
| Prescription medication cost | Lognormal, mean (sd) | 23.09 (4.30) |
| Proportion that go to the ED | Beta-Pert (min, mode, max) | 0, 0.06, 0.13 |
| ED visit charge^b^ | Normal, mean (sd)^b^ | 426.06 (19.10) |
| Proportion that miss work | Beta-Pert (min, mode, max) | 0, 0.06, 0.13 |
| Days of work missed | Triangular (min, mode, max) | 0, 1.67, 4.54 |
| **Ear illness ^a^** |  |  |
| Proportion that go to doctor | Beta-Pert (min, mode, max) | 0.93, 0.97, 1.00 |
| Proportion that take OTC | Beta-Pert (min, mode, max) | 0.22, 0.33, 0.45 |
| OTC medication cost | Lognormal, mean (sd) | 6.23 (1.26) |
| Proportion that take prescription | Beta-Pert (min, mode, max) | 0.81, 0.88, 0.96 |
| Prescription medication cost | Lognormal, mean (sd) | 16.02 (3.30) |
| Proportion that go to the ED | Beta-Pert (min, mode, max) | 0.03, 0.12, 0.19 |
| ED visit charge^b^ | Normal, mean (sd)^b^ | 467.30 (10.40) |
| Proportion that miss work | Beta-Pert (min, mode, max) | 0.03, 0.10, 0.17 |
| Days of work missed | Triangular (min, mode, max) | 0.56, 1.14, 1.72 |
| **Skin illness ^a^** |  |  |
| Proportion that take OTC | Beta-Pert (min, mode, max) | 0.22, 0.38, 0.53 |
| OTC medication cost | Lognormal, mean (sd) | 7.86 (2.10) |
| Proportion that take prescription | Beta-Pert (min, mode, max) | 0.58, 0.73, 0.87 |
| Prescription medication cost | Lognormal, mean (sd) | 19.22 (3.77) |
| Proportion that go to the ED | Beta-Pert (min, mode, max) | 0.00, 0.05, 0.12 |
| ED visit charge^b^ | Normal, mean (sd)^b^ | 459.38 (10.90) |
| Proportion that miss work | Beta-Pert (min, mode, max) | 0.00, 0.05, 0.12 |
| Days of work missed | Triangular (min, mode, max) | 0.00, 1.50, 7.85 |
| **Doctor Visit (Level 3) GI/Respiratory illness ^c^** |  |  |
| Cost for new patients | Normal, mean (sd) ^d^ | 114.47 (187.00) |
| Cost for existing patients | Normal, mean (sd) ^d^ | 78.00 (19.51) |
| **Doctor Visit (Level 2) Eye/Ear/Skin illness ^c^** |  |  |
| Cost for new patients | Normal, mean (sd) ^d^ | 96.00 (27.31) |
| Cost for existing patients | Normal, mean (sd) ^d^ | 58.00 (14.83) |
| ED professional fee (Level 3) GI/Respiratory illness | Normal, mean (sd) ^d^ | 180.00 (47.60) |
| ED professional fee (Level 2) Eye/Ear/Skin illness | Normal, mean (sd) ^d^ | 107.00 (28.87) |

^a^derived from NEEAR study (Wade et al. 2006; Wade et al. 2008; Wade et al. 2010).

^b^Charge for ED visit, not adjusted for CCR

^c^ (PMIC 2007)

^d^Normal distributions were bound at 0

Table S8: Parameters used to estimate the cost of severe illness

| ***Campylobacter*** | **Distribution** | **Parameters** |
| --- | --- | --- |
| ED Charge^a^ | Normal, mean (sd)^c^ | 1,334.29 (92.88) |
| Hospital Cost^b^ | Normal, mean (sd)^c^ | 5,547.03 (322.20) |
| Length of Stay^d^ | Triangular (min, mode, max) | 3.39, 3.59, 3.81 |
| Days of work missed^d^ | Point estimate | 6.75 |
| ***Cryptosporidium*** |  |  |
| ED Charge^a^ | Normal, mean (sd)^c^ | 1,513.90 (236.42) |
| Hospital Cost^b^ | Normal, mean (sd)^c^ | 6,699.30 (1,044.86) |
| Length of Stay^d^ | Triangular (min, mode, max) | 3.97, 4.93, 5.89 |
| Days of work missed^d^ | Point estimate | 7.60 |
| ***E. coli 0157H7*** |  |  |
| ED Charge^a^ | Normal, mean (sd)^c^ | 1,085.07 (75.31) |
| Hospital Cost^b^ | Normal, mean (sd)^c^ | 7,244.61 (946.87) |
| Length of Stay^d^ | Triangular (min, mode, max) | 3.92, 4.46, 5.00 |
| Days of work missed^d^ | Point estimate | 6.75 |
| ***Giardia*** |  |  |
| ED Charge^a^ | Normal, mean (sd)^c^ | 1,331.32 (149.77) |
| Hospital Cost^b^ | Normal, mean (sd)^c^ | 6,205.95 (644.23) |
| Length of Stay^d^ | Triangular (min, mode, max) | 3.28, 3.93, 4.60 |
| Days of work missed^d^ | Point estimate | 7.60 |
| ***Leptospira*** |  |  |
| ED Charge^a^ | Normal, mean (sd)^c^ | 1,797.20 (372.44) |
| Hospital Cost^b^ | Normal, mean (sd)^c^ | 12,064.55 (3,319.52) |
| Length of Stay^d^ | Triangular (min, mode, max) | 4.02, 5.95, 7.88 |
| Days of work missed^d^ | Point estimate | 10.00 |
| ***Naegleria fowleri*** ^e^ |  |  |
| ED Charge^a^ | Normal, mean (sd)^c^ | 1,967.20 (146.32) |
| Hospital Cost^b^ | Normal, mean (sd)^c^ | 20,456.73 (963.72) |
| Length of Stay^d^ | Triangular (min, mode, max) | 10.00, 10.65, 11.30 |
| Days of work missed^d^ | Point estimate | 100% fatal |
| ***Norovirus*** |  |  |
| ED Charge^a^ | Normal, mean (sd)^c^ | 1,174.24 (193.43) |
| Hospital Cost^b^ | Normal, mean (sd)^c^ | 6,758.24 (1,737.04) |
| Length of Stay^d^ | Triangular (min, mode, max) | 3.17, 4.24, 5.31 |
| Days of work missed^d^ | Point estimate | 3.57 |
| ***Shigella*** |  |  |
| ED Charge^a^ | Normal, mean (sd)^c^ | 1,560.63 (193.53) |
| Hospital Cost^b^ | Normal, mean (sd)^c^ | 5,159.32 (431.68) |
| Length of Stay^d^ | Triangular (min, mode, max) | 3.32, 3.73, 4.15 |
| Days of work missed | Point estimate | 3.70 |
| **Unknown GI** |  |  |
| ED Charge^a^ | Normal, mean (sd)^c^ | 1,328.61 (46.52 ) |
| Hospital Cost^b^ | Normal, mean (sd)^c^ | 4,421.99 (96.24) |
| Length of Stay^d^ | Triangular (min, mode, max) | 2.72, 2.77, 2.83 |
| Days of work missed^d^ | Point estimate | 3.57 |
|  |  |  |
| **Doctor visit Level 4 (new)**^f^ | Normal, mean (sd)^c^ | 118.00 (31.21) |
| **Doctor visit Level 4 (established)** ^f^ | Normal, mean (sd)^c^ | 192.00 (54.62) |
| **ED professional fee** ^f^ | Normal, mean (sd)^c^ | 297.26 (48.95) |
| **Hospital initial fee (all pathogen, except NF and Vibrio)** ^f^ | Normal, mean (sd)^c^ | 218.39 (32.39) |
| **Hospital initial fee (NF and Vibrio)** ^f^ | Normal, mean (sd)^c^ | 261.22 (58.23) |
| **Subsequent care (hospitalization)** ^f^ | Normal, mean (sd)^c^ | 113.53 (19.88) |
| **Discharge (Hospital)**^f^  **(all pathogens except NF and Vibrio)** | Normal, mean (sd)^c^ | 127.96 (18.87) |
| **Discharge (Hospital) (NF and Vibrio)** ^f^ | Normal, mean (sd)^c^ | 156.46 (36.20) |
| **VSL (Millions of $)** ^g^ | Normal, mean (sd)^c^ | 7.6 (4.8) |

^a^Charge for ED visit, not adjusted for CCR (NEDS)

^b^Cost for Hospital, adjusted for CCR, not including professional fees (NIS)

^c^Normal distributions were bound at 0

^d^ (ERS (Economic Research Service) 2014)

^e^ Bacterial Meningitis (ICD-9-CM 320.0) used as a surrogate

^f^ (PMIC 2007)

^g^ (US EPA 2010)

*Vibrio* spp. estimated from (ERS, 2014), no variability

|  |  | Age categories | | | |
| --- | --- | --- | --- | --- | --- |
|  |  | **0-10** | **11-19** | **20-54** | **55+** |
| GI | Mild (%) | 30.3 | 13.8 | 51.9 | 4.0 |
|  | Moderate (%) | 35.7 | 17.4 | 42.9 | 4.1 |
| Respiratory | Mild (%) | 36.1 | 16.1 | 45.7 | 2.0 |
|  | Moderate (%) | 48.5 | 16.2 | 32.3 | 3.1 |
| Eye | Mild (%) | 18.5 | 13.9 | 61.3 | 6.3 |
|  | Moderate (%) | 49.0 | 16.3 | 26.5 | 8.2 |
| Ear | Mild (%) | 24.5 | 15.6 | 57.1 | 2.8 |
|  | Moderate (%) | 50.8 | 13.4 | 34.3 | 1.5 |
| Skin | Mild (%) | 29.1 | 14.1 | 53.2 | 3.7 |
|  | Moderate (%) | 57.1 | 14.3 | 25.0 | 3.6 |

Table S9: Proportion of mild and moderate illnesses by age category

Table S10: Estimated number of outbreak cases, hospitalizations, and deaths due to water recreation (C90)

| **Pathogen** | **Number of Outbreak Cases**  **Mean (C90)** | **Percent Hospitalized ^a^**^b^ | **Number hospitalized**  **Mean (C90)** | **Percent died** **^a^**^b^ | **Number of deaths**  **Mean (C90)** |
| --- | --- | --- | --- | --- | --- |
| *Campylobacter* spp. | 130  (4-495) | 13.0-25.0 | 31.6  (1.1-123.5) | 0-0.4 | 0.4  (0.0-1.4) |
| *Cryptosporidium parvum* | 191  (27-570) | 13.0-25.0 | 61.7  (9.5-179.2) | 0-0.3 | 0.3  (0.0-0.7) |
| *Escherichia coli* O157:H7 | 252  (12-922) | 29.5-46.0 | 167.5  (7.8-647.2) | 0.5-0.8 | 2.7  (0.1-10.1) |
| *Giardia lamblia* | 28  (1-106) | 0-13.0 | 5.0  (0.1-18.2) | 0-0.1 | 0.1  (0.0-0.2) |
| *Leptospira* | 125  (3-479) | 30.0-65.2 | 113.7  (2.9-484.8) | 1.0-14.0 | 11.6  (0.3-45.2) |
| *Naegleria fowleri* | 4  (1-7) | 100.0 ^c^ | 4.0  (4.0-4.0) | 99.9-100 | 4.0  (4.0-4.0) |
| Norovirus^d^ | 880  (200-2,250) | 0.03 | 51.5  (12.9-123.6) | 0.01 | 0.2  (0.0-0.4) |
| *Shigella* spp. | 627  (76-1,952) | 13.9-22.0 | 246.1  (26.5-821.5) | 0.1-0.2 | 2.0  (0.2-6.5) |
| *Vibrio*  *parahaemolyticus* ^e^ | 22  (12-31) | 33.3-50.0 | 17.3  (9.9-24.9) | 0-4.8 | 0.6  (0.1-1.3) |
| *Vibrio alginolyticus* ^e^ | 38  (11-65) | 13.9-21.1 | 10.4  (4.3-23.8) | 0-4.2 | 1.2  (0.2-2.7) |
| *Vibrio* spp., other ^e^ | 8  (4-11) | 11.1-55.6 | 5.1  (2.2-8.8) | 5.2-20.0 | 1.0  (0.2-2.3) |
| *Vibrio vulnificus*^e^ | 30  (8-51) | 62.5-79.0 | 47.8  (14.2-83.0) | 5.0-23.5 | 9.1  (2.7-16.5) |
| *Vibrio cholera*^e^ | 4  (1-7) | 0-80.0 | 2.9  (0.6-6.1) | 0-10.0 | 0.5  (0.1-1.0) |
| Unknown GI illness | 87  (7-296) | 0.06–0.17 | 0.93  (0.24-1.85) | 0.001-0.002 ^f^ | 0.03  (0.0-0.01) |
| **Total** | **2,417**  **(1,097-4,624)** | **--** | **778**  **(333-1,696)** | **--** | **32**  **(16-67)** |

^a^Doubled to account for underdiagnoses (Mead et al. 1999; Scallan et al, 2011a)

^b^According to: Dechet et al., 2008; Dziuban et al. 2006; Goarant et al. 2009; Hlavsa et al. 2011; Hlavsa et al. 2014; Mead et al., 1999; Pond, 2005; Scallan et al. 2011a; Yoder et al. 2008; Yoder et al. 2010;

^c^Assumed to be 100%

^d^No variation in hospitalization or death rates

^e^Proportion hospitalized and died based on COVIS (CDC 2012) data presented in WBDOSS (Dziuban et al. 2006; Hlavsa et al. 2011; Yoder et al. 2008)

^f^Estimated from 2007 NIS

Table S11: Total cost of mild waterborne illness; mean (C90)

| **Outcome** | **Total cost in Millions of US dollars^a^** | | | **US dollars** |
| --- | --- | --- | --- | --- |
|  | Meds^b^ | Lost Productivity | Total Cost | **Cost per case** |
| GI | 148.5  (102.9-183.8) | 366.0  (255.1-473.0) | 461.3  (342.2-586.5) | **10.66**  **(9.53-11.65)** |
| Respiratory | 45.9  (6.4-83.5) | 45.2  (7.3-97.0) | 91.1  (13.7-179.1) | **11.45**  **(10.88-13.56)** |
| Eye | 37.0  (0.2-74.7) | -- | 61.4  (0.4-123.2) | **9.07**  **(8.01-10.06)** |
| Ear | 17.3  (6.2-26.6) | 25.7  (7.1-50.3) | 43.0  (14.7-73.3) | **8.27**  **(5.28-11.80)** |
| Skin | 46.7  (24.3-62.4) | -- | 46.7  (24.3-62.4) | **4.09**  **(3.24-4.50)** |
| Total Cost of Mild Illness | 295.4  (213.2-355.1) | 461.3  (342.2-586.5) | 756.7  (565.3-928.3) | **9.50**  **(8.62-10.34)** |

^a^Estimated costs (in millions of 2007 dollars) may not sum to estimated totals due to rounding

^b^Combined OTC and prescription medication costs

Table S12: Total cost of moderate waterborne illness; mean (C90)

| **Outcome** | **Total cost in Millions of US dollars^a^** | | | | | **US dollars** |
| --- | --- | --- | --- | --- | --- | --- |
|  | Meds ^b^ | Doctor visit | ED visit | Lost Productivity | Total Cost | **Cost per case** |
| GI | 78.3  (47.0-109.0) | 308.9  (189.7-453.8) | 775.5  (499.2-1,072.0) | 132.1  (82.6-189.0) | 1,294.8  (875.9-1,733.0) | **343.42**  **(290.20-401.93))** |
| Respiratory | 24.8  (3.3-45.3) | 108.8  (14.4-207.4) | 65.6  (8.4-126.5) | 35.5  (4.5-67.3) | 234.7  (31.6-426.1) | **185.73**  **(151.15-217.41)** |
| Eye | 17.3  (0.1-36.1) | 50.6  (0.31-111.3) | 14.3  (0.1-36.4) | 9.8  (0.1-40.2) | 91.9  (0.6-205.6) | **117.08**  **(91.99-153.65)** |
| Ear | 27.1  (9.1-42.4) | 104.4  (35.1-170.8) | 62.3  (19.7107.9) | 23.8  (6.7-43.5) | 217.6  (077.1-337.4) | **130.38**  **(101.36-158.12)** |
| Skin | 12.0  (5.8-18.8) | 42.2  (20.4-68.9) | 7.2  (2.0-17.7) | 10.8  (0.6-31.8) | 61.5  (36.5-118.6) | **111.73**  **(80.23-148.93)** |
| Total Cost of Moderate Illness | 159.5  (107.7-199.0) | 6150  (427.3-803.3) | 924.9  (631.0-1,226.7) | 201.1  (146.2-286.5) | 1,900.5  (1,393.6-2,400.6) | **202.30**  **(202.30-277.15)** |

^z^Estimated costs (in millions of 2007 dollars) may not sum to estimated totals due to rounding

^b^Combined OTC and prescription medication

Table S13: Total cost of severe waterborne illness; mean (C90)

|  | **Total cost in Millions of US dollars ^a^** | | | | | | | |
| --- | --- | --- | --- | --- | --- | --- | --- | --- |
|  | Meds | Doctor/ED visit | Hospital visit | Lost Productivity^b^ | Death | Sequelae | Total Cost | **Cost per case** |
| *Campylobacter* spp. | 0.00^c^  (0.00^c^ -0.01) | 0.02  (0.00^c^-0.06) | 0.19  (0.0^1^-0.73) | 0.01  (0.00^c^-0.06) | 1.98  (0.50-12.40) | 0.01  (0.00^c^-0.05) | 2.21  (0.07–13.24) | **0.07**  **(0.00** **^c^ –0.21)** |
| *C. parvum* | 0.00^c^  (0.00^c^ -0.01) | 0.03  (0.00^c^-0.10) | 0.39  (0.06-1.13) | 0.03  (0.00^c^-0.09) | 0.03  (0.03-6.17) | **--** | 0.47  (0.15–7.31) | **0.01**  **(0.01** ^c^ **–0.09)** |
| *E. coli* O157:H7 | 0.01  (0.00^c^ -0.03) | 0.07  (0.00^c^-0.27) | 1.10  (0.58-4.34) | 0.07  (0.00^c^-0.26) | 19.21  (0.55-78.09) | 0.36  (0.02-1.41) | 20.81  (0.74–83.81) | **0.14**  **(0.03–0.25)** |
| *Giardia lamblia* | 0.00^c^  (0.00^c^ -0.00^c^) | 0.00^c^  (0.00^c^-0.01) | 0.03  (0.00^c^-0.11) | 0.00^c^  (0.00 ^c^ -0.01) | 0.43  (0.00^c^-1.71) | **--** | 0.46  (0.01–1.82) | **0.09**  **(0.03–0.26)** |
| *Leptospira* | 0.01  (0.00^c^-0.02) | 0.07  (0.00^c^-0.25) | 1.51  (0.04-5.73) | 0.08  (0.00^c^-0.28) | 66.57  (1.17-384.81) | **--** | 68.22  (1.29–390.80) | **0.55**  **(0.12–2.07)** |
| *N. fowleri* | 0.00^c^  (0.00^c^ -0.00^c^) | 0.00^c^  (0.00^c^ -0.00^c^) | 0.08  (0.08-0.09) | **-**^d^ | 30.41  (6.15-62.82) | **--** | 30.50  (6.23–62.91) | **7.6**  **(1.6–15.7)** |
| Norovirus | 0.00^c^  (0.00^c^-0.01) | 0.02  (0.00^c^-0.06) | 0.36  (0.07-0.95) | 0.01  (0.00^c^ -0.03) | 1.34  (0.14-4.26) | **--** | 1.74  (0.30–5.18) | **0.03**  **(0.01–0.06)** |
| *Shigella* spp. | 0.01  (0.00^c^-0.04) | 0.13  (0.01-0.39) | 1.30  (0.15-3.89) | 0.06  (0.00^c^-0.18) | 15.28  (0.92-54.6) | 0.01  (0.00^c^-0.04) | 16.79  (1.28–58.66) | **0.07**  **(0.02–0.14)** |
| *V. parahaemolyticus* | 0.00^c^  (0.00^c^ -0.00^c^) | 0.01  (0.00^c^-0.01) | 0.16  (0.09-0.24) | 0.01  (0.01-0.02) | 2.60  (0.35-13.38) | **--** | 2.78  (0.51–13.59) | **0.17**  **(0.03–0.76)** |
| *V. alginolyticus* | 0.00^c^  (0.00^c^ -0.00^c^) | 0.00^c^  (0.00^c^-0.01) | 0.10  (0.04-0.22) | 0.01  (0.00^c^ -0.02) | 7.0  (0.77-28.07) | **--** | 7.11  (0.87–28.26) | **0.68**  **(0.09–1.91)** |
| *Vibrio* spp., other | 0.00^c^  (0.00^c^ -0.00^c^) | 0.00^c^  (0.00^c^ -0.00^c^) | 0.05  (0.02-0.08) | 0.00^c^  (0.00^c^-0.01) | 5.93  (0.74-23.02) | **--** | 5.98  (0.78–23.10) | **1.18**  **(0.18–4.81)** |
| *V. vulnificus* | 0.00^c^  (0.00^c^ -0.00^c^) | 0.03  (0.01-0.05) | 1.46  (0.47-2.57) | 0.03  (0.01-0.05) | 70.98  (8.19-184.96) | **--** | 72.49  (9.22–187.19) | **1.54**  **(0.30–3.34)** |
| *V. cholera* | 0.00^c^  (0.00^c^ -0.00^c^) | 0.00^c^  (0.00^c^ -0.00^c^) | 0.03  (0.00^c^-0.06) | 0.00^c^  (0.00^c^ -0.00^c^) | 3.81  (0.41-10.02) | **--** | 3.84  (0.42–10.06) | **1.43**  **(0.20–5.04)** |
| Unknown GI | 0.00^c^  (0.00^c^ -0.00^c^) | 0.00^c^  (0.00^c^ -0.03) | 0.04  (0.01-0.29) | 0.00^c^  (0.00^c^-0.02) | 0.23  (0.02-1.74) | **--** | 0.28  (0.03–2.05) | **0.03**  **(0.01–0.06)** |
| Total Cost of Severe Symptoms | 0.04  (0.01-0.08) | 0.37  (0.15-0.85) | 6.79  (3.19-14.07) | 0.31  (0.13-0.69) | 225.79  (103.55-599.07) | 0.38  (0.03-1.44) | 233.68  (108.49-613.95) | **0.30**  **(0.16–0.78)** |

^a^Estimated costs (in millions of 2007 dollars) may not sum to estimated totals due to rounding

^b^Estimated only for nonfatal cases

^c^Estimated cost <.01 million

^d^No lost productivity estimated, >99.9% cases are fatal

**References**

CDC (Centers for Disease Control and Prevention). 2012. Cholera and other vibrio illness surveillance overview. Atlanta, Georgia:US Department of Health and Human Services.

Cordell HK. 2012. Outdoor recreation trends and futures: A technical document supporting the forest service 2010 RPA assessment. Asheville, NC:Department of Agriculture Forest Service.

Dechet AM, Patricia AY, Koram N, Painter J. 2008. Nonfoodborne vibrio infections: An important cause of morbidity and mortality in the United States, 1997--2006. Clinical Infectious Diseases 46:970-976.

DeFlorio-Barker S, Wade TJ, Jones RM, Friedman LS, Wing C, Dorevitch S. 2016b. Estimated costs of sporadic gastrointestinal illness associated with surface water recreation: A combined analysis of data from neear and cheers studies. Environ Health Perspect.

Dorevitch S, Pratap P, Wroblewski M, Hryhorczuk DO, Li H, Liu LC, et al. 2012a. Health risks of limited-contact water recreation. Environmental Health Perspectives 120:192-197.

Dorevitch S, DeFlorio-Barker S, Jones R, Liu L. 2015. Water quality as a predictor of gastrointestinal illness following incidental contact water recreation. Water Research 83:94-103.

Dziuban EJ, Beach MJ, Liang JL, Craun GF, Hill V, Yu PA, et al. 2006. Surveillance for waterborne disease and outbreaks associated with recreational water--United States, 2003-2004. MMWR 55.

ERS (Economic Research Service). 2014. Cost estimates of foodborne illnessess.U.S. Department of Agriculture.

Frenzen PD, Drake A, Angulo FJ, The Emerging Infections Program Foodnet Working G. 2005. Economic cost of illness due to escherichia coli:O157 infections in the United States. Journal of Food Protection 68:2623-2630.

Frenzen PD. 2007. An online cost calculator for estimating the economic cost of illness due to shiga toxin-producing *E. coli* (stec) 0157 infections.United States Department of Agriculture, Economic Research Service.

Frenzen PD. 2008. Economic cost of guillain-barre syndrome in the United States. Neurology 71:21-27.

Glennas A, Kvien TK, Melby K, OverbÃ¸o A, Andrup O, Karstensen B, et al. 1994. Reactive arthritis: A favorable 2 year course and outcome, independent of triggering agent and hla-b27. The Journal of rheumatology 21:2274-2280.

Goarant C, Laumond-Barny S, Perez J, Vernel-Pauillac F, Chanteau S, Guigon A. 2009. Outbreak of leptospirosis in new caledonia: Diagnosis issues and burden of disease. Trop Med Int Health 14:926-929.

Hlavsa MC, Hilborn ED, Wade TJ, Beach MJ, Yoder JS, Roberts VA, et al. 2011. Surveillance for waterborne disease outbreaks and other health events associated with recreational water --- United States, 2007-2008. MMWR 60.

Hlavsa MC, Roberts VA, Kahler AM, Hilborn ED, Wade TJ, Backer LC, et al. 2014. Recreational water-associated disease outbreaks--United States, 2009-2010. MMWR 63:6.

Hoffmann S, Batz MB, Morris Jr JG. 2012. Annual cost of illness and quality-adjusted life year losses in the United States due to 14 foodborne pathogens. Journal of Food Protection 75:1292-1302.

Mead PS, Slutsker L, Dietz V, McCaig LF, Bresee JS, Shapiro C, et al. 1999. Food-related illness and death in the United States. Emerging infectious diseases 5:607.

PMIC (Practice Management Information Corporation). 2007. Medical fees in the United States: Nationwide charges for medicine, surgery, laboratory, radiology and allied health services. Los Angeles, CA:Practice Management Information Corp.

Pond K. 2005. Water recreation and disease: Plausibility of associated infections: Acute effects, sequelae, and mortality:IWA publishing.

Roberts JD, Silbergeld EK, Graczyk T. 2007. A probabilistic risk assessment of cryptosporidium exposure among Baltimore urban anglers. J Toxicol Eviron Health A 70:1568-1576.

Scallan E, Hoekstra RM, Angulo FJ, Tauxe RV, Widdowson MA, Roy SL, et al. 2011. Foodborne illness acquired in the United States--major pathogens. Emerging infectious diseases 17:7-15.

Scharff RL. 2011. Economic burden from health losses due to foodborne illness in the United States. J Food Protect 75:123-131.

Townes JM, Deodhar AA, Laine ES, Smith K, Krug HE, Barkhuizen A, et al. 2008. Reactive arthritis following culture-confirmed infections with bacterial enteric pathogens in minnesota and oregon: A population-based study. Annals of the rheumatic diseases 67:1689-1696.

US EPA USEPA. 2010. Guidelines for preparing economic analysis. Washington, DC:Office of Policy, U.S. Environmental Protection Agency.

Wade TJ, Calderon RL, Sams E, Beach M, Brenner KP, Williams AH, et al. 2006. Rapidly measured indicators of recreational water quality are predictive of swimming-associated gastrointestinal illness. Environmental Health Perspectives 114:24-28.

Wade TJ, Calderon RL, Brenner KP, Sams E, Beach M, Haugland R, et al. 2008. High sensitivity of children to swimming-associated gastrointestinal illness: Results using a rapid assay of recreational water quality. Epidemiology 19:375-383.

Wade TJ, Sams E, Brenner KP, Haugland R, Chern E, Beach M, et al. 2010. Rapidly measured indicators of recreational water quality and swimming-associated illness at marine beaches: A prospective cohort study. Environmental Health 9:66.

Wade TJ, Sams E, Brenner KP, Haugland R, Chern E, Beach M, et al. 2010a. Rapidly measured indicators of recreational water quality and swimming-associated illness at marine beaches: A prospective cohort study. Environ Health 9:66.

Yoder JS, Blackburn BG, Craun GF, Hill V, Levy DA, Chen N, et al. 2004. Surveillance for waterborne-disease outbreaks associated with recreational water--United States, 2001-2002. MMWR 53:1-22.

Yoder JS, Hlavsa MC, Craun GF, Hill V, Roberts V, Yu PA, et al. 2008. Surveillance for waterborne disease and outbreaks associated with recreational water use and other aquatic facility-associated health events--United States, 2005-2006. MMWR 57.

Yoder JS, Eddy BA, Visvesvara GS, Capewell L, Beach MJ. 2010. The epidemiology of primary amoebic meningoencephalitis in the USA, 1962-2008. Epidemiol Infect 138:968-975.
